# IPC training in Sierra Leone- ICAN's role in fighting Ebola

**DOI:** 10.1186/2047-2994-4-S1-O12

**Published:** 2015-06-16

**Authors:** S Mehtar, B Hakizimana

**Affiliations:** 1 UIPC, Stellenbosch University, Cape Town, Infection Control Africa Network, Cape Town, South Africa

## Introduction

Thousands of people have been affected by Ebola; the crude mortailty rate is estimated at around 53%. Responding to WHO, the Infection Control Africa Network (ICAN) has been at the forefront of fighting Ebola since April 2015. Two training courses were presented between February and mid March 2015 in Sierra Leone funded by the WHO & CDC.

## Objectives

The training was to ensure that evidence based IPC practices were put in place to counteract rituals, myths and superstition which surrounded the community and healthcare workers (HCW) with increasing spread of Ebola; to clear the confusion created by the several organisations teaching different methods of IPC.

## Methods

The Basic Ebola IPC course registered at Stellenbosch University, was delivered to 60 and 75 students for the WHO and CDC respectively. A pre and post assessment examination measured the level of knowledge gained. Didactic lectures were delivered in the morning and practical work in the afternoon. The WHO guidelines on Ebola were used as a basis for training these HCW to function at National and District level.

A two week course was CDC funded. The first week was the Ebola IPC course, the second was CDC material based on the National SOP for Ebola IPC in Sierra Leone to train the trainers. Presentation to their peers were marked for presentation, content, clarity, material, time keeping and scientific content.

Both the training programmes were anonymously evaluated by the students each day.

## Results

Both groups were experienced but needed support. The average increase between pre and post test knowledge was 27% for both sets of students; starting at 37% post test average was 87%. An increased level of confidence, presentation/ communication skills was 32%. By the end, evidence and risk assessment were driving their decision making. Course evaluation averaged between 87% and 92%.

## Conclusion

The training provided by ICAN funded by the WHO and CDC had a major impact on the outcome of IPC in Sierra Leone. The students are confident in dealing with Ebola, carrying out appropriate risk assessments and protecting themselves, their colleagues and their patients.

## Disclosure of interest

None declared.

